# Professional Secrecy and Public Justice

**Published:** 1915-07-31

**Authors:** 


### The Hospital

The Workers' Newspaper of Administrative Medicine and Institutional Life, Administration, National Insurance and Health.

No. 1520, Vol. LVIII. SATURDAY, JULY 31, 1915. PRICE ONE PENNY

### PROFESSIONAL SECRECY AND PUBLIC JUSTICE.

The relations which exist between the sick man and his medical adviser impose upon the latter as an obligation of honour the duty of treating as sacred the confidences which have been committed to his keeping. To break such confidences means, and ought to mean, personal discredit, and if harmful consequences follow, material damages may well be awarded against the offending practitioner in a court of law. The only safe course for the doctor who would avoid either or both of these results is to cultivate the habit of not talking about his patients at all. To attempt to distinguish between the important and the unimportant is here impossible; and what seems at the moment well-intentioned information may in the event prove to be highly mischievous. It is not the man who cultivates a still tongue who will give away his patients' secrets, while a deliberate breach of confidence must be rare. The danger and risk arise from an easy, friendly, careless habit which seems to be harmless until it produces the catastrophe that a cultivated and deliberate silence and reserve can never suffer. Up to this point the line of professional duty is easy to define. There may be difficulties in dealing with benevolent inquiries from kindly or inquisitive neighbours, and in deciding what amount of information is due to a person holding some particular relation to the patient, but these are to be met by tact and a little kindly common sense. The principle remains clear, and if there is doubt about its application in any individual instance, the tendency must be to a rigid rather than to a loose construction. What the doctor learns in his professional capacity about the patient and his affairs is sacred, and if in any given instance the rule is broken, the onus of proving that there were special circumstances which justified the breach lies upon the practitioner who has not kept faith. All this is not some pedantic professional etiquette or the lofty claim to a superior code of honour. On the contrary, the practice has as its sanction the strictly utilitarian considerations that sick people will not take medical advice if their secrets are likely to be betrayed; that efficient treatment is likely to be hindered by want of frankness in the patient; and that it is in the public interest that the victims of disease should, at an early stage of their maladies and without reserve, be in the hands of competent medical practitioners rather than they should become the subjects of self-medication or the sport of amateur prescribers or the victims of quacks.

Now and again in medical practice there arises a situation in which the rule here discussed is liable to be challenged. Such a situation exists when the information obtained by the practitioner reveals more or less clearly the commission of a crime, and, to take a specific instance, the crime of abortion. Not very infrequently this position comes under review in a court of law. A woman dies after a premature delivery, evidences of improper interference are revealed on necropsy, an accused person is tried for the act, and the action of the doctor who was called in to see the woman in her last illness is commented on by the Judge. This is exactly what happened a few months ago at the Birmingham Assizes, and the Judge remarked adversely on the fact that the medical men concerned had neither given information to the police nor taken steps to secure that the woman made a statement capable of being utilised as legal evidence. It would be possible to quote other legal *obiter dicta* not altogether in harmony with these censures, but neither the one conclusion nor the other can be claimed as a definite legal finding. This could only be secured, we presume, by a verdict and judicial decision in a case where a medical man with the above experience was indicted in a trial for criminal abortion as "an accessory after the fact," since remarks from the Bench have judicial efficacy in their relation not to general propositions but to particular instances. Still it cannot but be an anxious matter for medical practitioners to know what exactly is their duty in the circumstances here contemplated, and the anxiety is sharpened by the open announcement of the opinion of one of the judges.

Quite properly, in these circumstances, some of the professional organisations to which the individual practitioner looks for guidance have reconsidered the question, and have issued a deliberate opinion on it. We refer to the recent pronouncements made by the Royal College of Physicians and by the British Medical Association. It is satisfactory to find these authorities in substantial agreement, to know that they speak under legal advice, and to learn that a practitioner is under no legal obligation to announce to the police that he knows or has reason to believe that criminal abortion has been practised on his patient. He may, especially if the woman seems likely to die, advise her to make a statement which can be used as evidence against the person who performed the operation, but no further duty can be claimed at his hands, though, of course, in the event of a fatal issue he would refuse a certificate of the cause of death. In a word, the general obligation to respect the confidence of the patient and to make no disclosure without her consent remains. The detailed decisions of the two bodies above mentioned should be carefully studied and preserved, for they are of supreme importance. Indications are not wanting that an effort will be made to impose on the profession increased responsibilities in this direction. These may have to be met by the most strenuous opposition, and this may fail unless the professional position can be fully justified in the opinion of the public. Medical practitioners are not, and do not intend to be, agents of the detective police. They are the trusted and confidential advisers of their patients, and in this capacity, while, of course, they will organise no measures for the concealment of crime, they must, in the public interest, claim the right to justify this confidence and to refuse obligations which are at variance with it.

